# Gene Expression Profiling in the Thiamethoxam Resistant and Susceptible B-biotype Sweetpotato Whitefly, *Bemisia tabaci*

**DOI:** 10.1673/031.012.4601

**Published:** 2012-03-30

**Authors:** Wen Xie, Xin Yang, Shao-Ii Wang, Qing-jun Wu, Ni-na Yang, Ru-mei Li, Xiaoguo Jiao, Hui-peng Pan, Bai-ming Liu, Yun-tao Feng, Bao-yun Xu, Xu-guo Zhou, You-jun Zhang

**Affiliations:** ^1^ Department of Plant Protection, Institute of Vegetables and Flowers, Chinese Academy of Agricultural Sciences, Beijing, 100081, China; ^2^ Department of Entomology, University of Kentucky, Lexington, KY 40546-0091, USA

**Keywords:** insecticide resistance, quantitative real-time PCR, NAD-dependent methanol dehydrogenase, sap-sucking insect, suppression subtractive hybridization

## Abstract

Thiamethoxam has been used as a major insecticide to control the B-biotype sweetpotato whitefly, *Bemisia tabaci* (Gennadius) (Hemiptera: Aleyrodidae). Due to its excessive use, a high level of resistance to thiamethoxam has developed worldwide over the past several years. To better understand the molecular mechanisms underlying this resistance in *B. tabaci*, gene profiles between the thiamethoxam-resistant and thiamethoxam-susceptible strains were investigated using the suppression subtractive hybridization (SSH) library approach. A total of 72 and 52 upand down-regulated genes were obtained from the forward and reverse SSH libraries, respectively. These expressed *sequence* tags (ESTs) belong to several functional categories based on their gene ontology annotation. Some categories such as cell communication, response to abiotic stimulus, lipid particle, and nuclear envelope were identified only in the forward library of thiamethoxam-resistant strains. In contrast, categories such as behavior, cell proliferation, nutrient reservoir activity, sequence-specific DNA binding transcription factor activity, and signal transducer activity were identified solely in the reverse library.

To study the validity of the SSH method, 16 differentially expressed genes from both forward and reverse SSH libraries were selected randomly for further analyses using quantitative realtime PCR (qRT-PCR). The qRT-PCR results were fairly consistent with the SSH results; however, only 50% of the genes showed significantly different expression profiles between the thiamethoxam-resistant and thiamethoxam-susceptible whiteflies. Among these genes, a putative NAD-dependent methanol dehydrogenase was substantially over-expressed in the thiamethoxamresistant adults compared to their susceptible counterparts. The distributed profiles show that it was highly expressed during the egg stage, and was most abundant in the abdomen of adult females.

## Introduction

The sweet potato whitefly, *Bemisia tabaci* (Gennadius) (Hemiptera: Aleyrodidae), is one of the most widely distributed agricultural pests worldwide (Perring 2001), attacking agronomic, horticultural, and ornamental crops in subtropical and tropical agriculture, as well as in greenhouse production systems (Oliveira et al. 2001). It was first identified in China in the mid-1990s and then spread into more than 20 provinces within a very short time frame (Luo et al. 2002; Chu et al. 2005; Zhang et al. 2005; Chu et al. 2006). The phloem-feeding whitefly has caused severe crop losses through direct feeding, excretion of honeydew (which favors sooty mold development), and transmission of plant viruses (Jones 2003).

Due to its severe damages, *B. tabaci* has been controlled predominantly with chemical insecticides. However, as a result of extensive application of synthetic insecticides, *B. tabaci* has developed a high degree of resistance to a wide range of insecticides, including carbamates, organophosphates, pyrethroids, insect growth regulators (IGRs), and neonicotinoids (Horowitz et al. 1988; Prabhaker et al. 1988; Horowitz et al. 1999; Nauen et al. 2002; Ahmad et al. 2002; Kranthi et al. 2002; Ma et al. 2007; Erdogan 2008; Roditakis 2009; Wang et al. 2010). Neonicotinoid insecticides are generally considered systemic and have excellent efficacy, long-lasting residual activity, and favorable safety profiles. For example, thiamethoxam, discovered and developed by the Novartis Crop Protection (www.novartis.com), has played a crucial role in controlling *B. tabaci* and many other sapsucking insect pests in China since its introduction in 2000. A high level of resistance to thiamethoxam (100- and 900fold), however, has already been reported in B- and Q-biotype of *B. tabaci* strains from Israel and Spain, respectively (Rauch and Nauen 2003; Horowitz et al. 2004). In China, both biotypes have developed high levels of resistance to imidacloprid and thiamethoxam in the field (Wang et al. 2010).

In general, the safety and effectiveness of neonicotinoids have been attributed to their high affinity to nicotinic acetylcholine receptors (nAChRs). Consequently, resistance to neonicotinoids initially focused on the mutations in nAChRs (Liu et al. 2009). In addition, recent studies revealed that resistance of neonicotinoids in *B. tabaci* could be associated with an enhanced oxidative detoxification by cytochrome P450 monooxygenases (Karunker et al. 2008; Wang et al. 2009). Through biochemical characterization of B-biotype thiamethoxamresistant strains, cytochrome P450 monooxygenase and carboxylesterase were found to be responsible for the thiamethoxam resistance in whiteflies (Feng et al. 2008, 2010).

Suppression subtractive hybridization (SSH) is a RNA-based method for identifying genes with unknown function, especially in species that lack primary genomic resources (Diatchenko et al. 1996; Lu and Wan 2008). This method has already been used to better understand the genetic basis of insecticide resistance, such as *Aedes aegypti* resistance to deltamethrin (Lertkiatmongkol et al. 2010) and *Nilaparvata lugens* resistance to triazophos (Bao et al. 2010). The SSH method has been applied to identify genes related to viral infection (Li et al. 2011) and heat-shock (Lü and Wang 2008) in *B. tabaci* as well. In this study, gene expression profiles between the thiamethoxam-resistant and thiamethoxam-susceptible *B. tabaci* were investigated by both SSH and qRT-PCR analyses. Combined results give us a unique perspective in regards to the development of neonicotine resistance in the B-biotype *B. tabaci*.

## Materials and Methods

### *Bemisia tabaci* strains

The B-biotype *B. tabaci* susceptible strain (TH-S) and resistant strain (TH-R) were the same populations as described previously (Feng et al. 2008, 2010). Before sample collection, a leaf-dip bioassay (Feng et al. 2008) was conducted to confirm that the resistance factor (LC_50_ (TH-R)/LC_50_ (TH-S)) was at least 70-fold. About 3000 adult whiteflies from TH-R were treated with 2000 mg/L thiamethoxam (∼LC_80_) to eliminate the heterozygous individuals. Then, the survivors were collected after 48 hours and designated as the TH-2000. A total of 300 TH-S and TH2000 adults, respectively, were collected, snap frozen in liquid nitrogen for three hours, and transferred to a -80 °C freezer for long-term storage. Different developmental stages, such as eggs, 3^rd^ instar larvae, and two-day-old unmated adult females, and various tissues including head, thorax, abdomen, and wing of a two-day-old unmated adult female were collected to study the distribution profiles of genes of interest.

### Total RNA isolation and reverse transcription

Total RNAs from both TH-2000 and TH-S adults were extracted using Trizol (Invitrogen, www.invitrogen.com) following manufacturer protocol. The resulting total RNA was resuspended in nuclease-free water, and quantified by the Nanodrop 2000 (Thermo Scientific, www.thermoscientific.com). The first-strand cDNA and ds-cDNA were synthesized using SMARTer™ PCR cDNA Synthesis Kit (Clontech, www.clontech.com) and later, the ds-cDNA were purified with QIAquick PCR Purification Kit (QIAGEN, www.quiagen.com).

### Construction of the SSH cDNA library

The SSH procedure was carried out using a PCR-Select™ cDNA Subtraction Kit (Clontech) following manufacturer protocol. A forward SSH library was constructed to isolate the up-regulated genes of the TH-2000 whitefly strain. The forward SSH library was used to identify clones in which the TH-2000 cDNA was used as the tester and the TH-S cDNA as the driver. In addition, a reverse SSH library was constructed to detect the down-regulated genes of the TH-2000 whitefly strain. The reverse SSH library was used to identify clones in which the TH-S cDNA was used as the tester and the cDNA from TH-2000 as the driver. After hybridization, the subtracted cDNA were ligated into the pGEM-T vector (Promega, www.promega.com) and transformed into *Escherichia coli* competent cells through electroporation.

### DNA sequencing and EST analysis

Positive clones were selected by conventional blue-white screening. White clones were randomly selected from both forward and reverse libraries. The positive clones were further validated by colony PCR using nested PCR primers provided in the kit. The resulting products were subjected to the direct sequencing with M13 primers. The vector sequences were removed through a Perl script and checked through Vec Screen (http://www.ncbi.nlm.nih.gov/VecScreen/VecScreen.html). Then, the remaining highquality EST sequences were analyzed in the GenBank non-redundant (nr) database with BLASTX. A sequence was considered as significantly matched when the E-value was < 10^-5^. Functional annotation was carried out in the Swiss-Prot (http://expasy.org/people/swissprot.html). Gene Ontology (GO) terms were extracted and counted using map2slim and Perl scripts.

### Quantitative real time PCR

Up to 150 (three biological replicates, n = 50) TH-S and TH-2000 adults, respectively, were collected for the qRT-PCR analysis. Approximately 0.5 µg of total RNA was used as a template to synthesize the first-strand cDNA using PrimerScript RT reagent Kit (Takara Bio Inc., www.takarabio.com) following manufacturer protocol. The resulting cDNA was diluted to a working concentration of 0.1µg/µL for the subsequent qRT-PCR analysis. To validate the differentially expressed genes detected by the SSH approach, 16 expressed ***sequence*** tags (ESTs) representing 11 putatively upregulated genes and five down-regulated genes were randomly selected. The qRT-PCR primers were designed using Primer3 (http://frodo.wi.mit.edu/primer3) (Table 1). The cycling parameters were as follows: 95 ° C for 30 sec, followed by 40 cycles of 95 °C for five sec and 62 °C for 34 sec, and ended with a melting curve analysis (65 °C to 95 °C in increments of 0.5 °C every five sec) to check for nonspecific product amplification. Relative gene expression was calculated using the 2^-ΔΔCt^ method, β-actin was used as the internal reference gene to eliminate sample-tosample variations in the initial cDNA samples.

**Figure 1.**
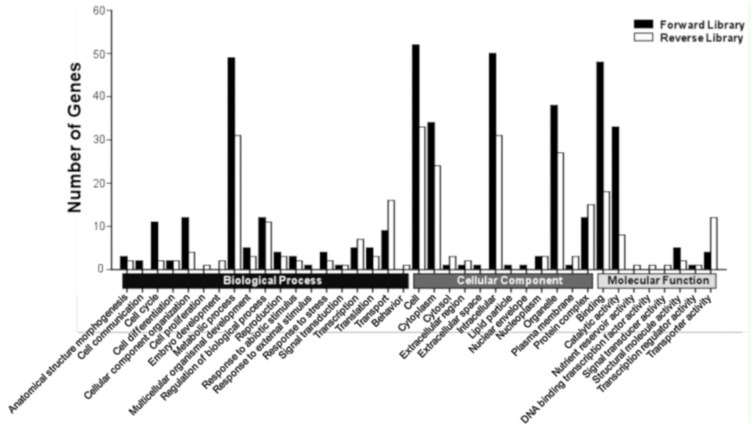
Gene Ontology (GO) classification of the differentially expressed expression ESTs in the forward and reverse SSH libraries. Based on the GO terms, the ESTs are categorized into putative functional groups. The black and white columns represent the up- and down-regulated genes from forward and reverse libraries, respectively. High quality figures are available online.

## Results

### Differential screening and EST sequencing

Based on the results of the differential screening, all 507 cDNA clones were randomly picked and sequenced from these two libraries. Specifically, 298 clones were from the forward library representing upregulated genes, and 209 clones were from the reverse library representing down-regulated genes. After trimming, 127 and 63 highquality ESTs from the forward and reverse library, respectively, were obtained.

**Figure 2.**
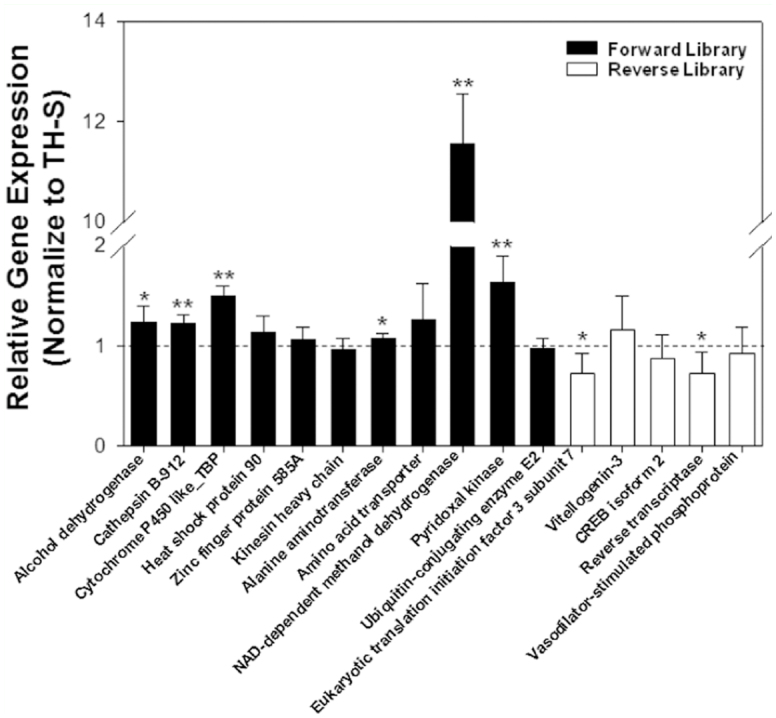
Quantitative real-time PCR validation. The gene expression level of 16 randomly selected ESTs, including 11 from SSH forward library (the black column) and five from SSH reverse library (the white column), was tested using qRT-PCR. The relative gene expression level in the resistant strains was normalized to the susceptible TH-S whiteflies. Data are presented as mean ± SE. Asterisks denote significant gene expression differences between the resistant and susceptible whiteflies, as determined by the pairwise *t*-tests (^*^ *p* < 0.05, ^**^ *p* < 0.01, LSD *t*-test). High quality figures are available online.

### Functional annotation

The BLASTX results showed that among the 127 clones from the SSH forward library, 72 ESTs (56.7%) had significant matches (Evalue < 10^-5^) to known or predicted genes in GenBank, and these clones could be assembled into 61 distinct sequences (Supplementary Table 1). For the 63 clones from the SSH reverse library, 52 ESTs (83.8 %) had significant matches (E-value < 10^-5^) with the database, among which 39 distinct sequences were identified (Table S2).

Based on the Gene Ontology (GO) terms, these distinct sequences were functionally annotated (Figure 1). Majority of the biological processes (such as cell cycle, metabolic process, response to external stimulus, response to stress, and transport), some cellular components (such as cell, plasma membrane, and protein complex), and some molecular functions (such as binding, catalytic activity, structural molecule activity, transcription regulator activity, and transporter activity) were presented in both libraries. Other GO terms, such as cell communication, response to abiotic stimulus, lipid particle, and nuclear envelope were identified only in the forward library. Vice versa, some GO terms such as behavior, cell proliferation, nutrient reservoir activity, sequence-specific DNA binding transcription factor activity, and signal transducer activity were identified only in the reverse library.

### qRT-PCR validation

**Figure 3.**
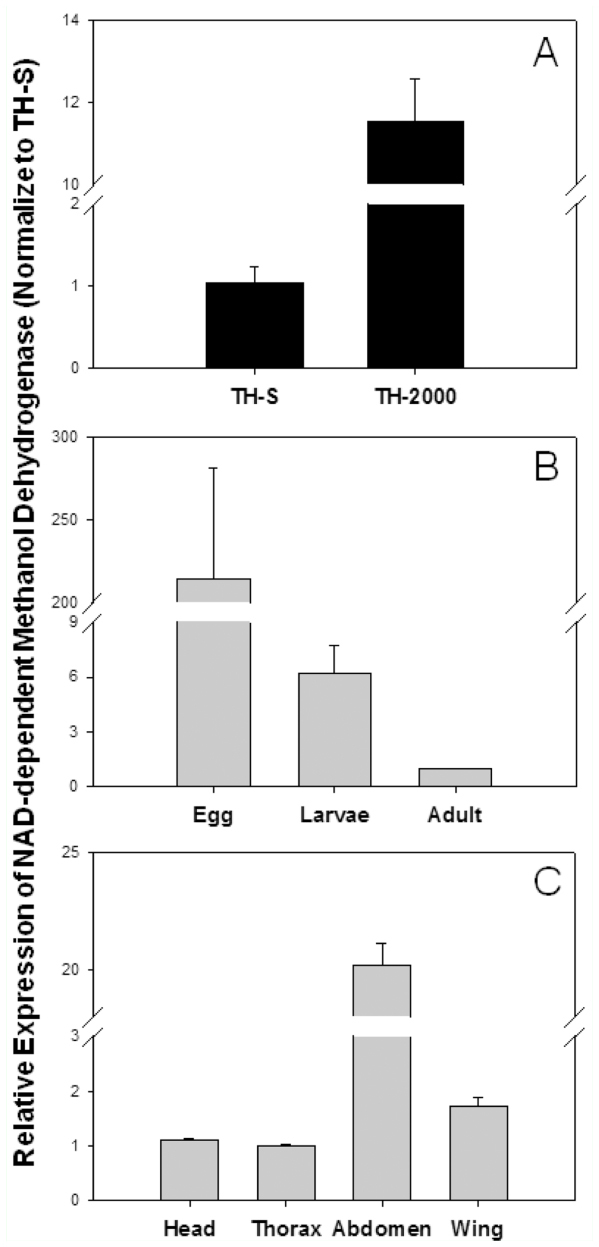
Gene expression profiles of a putative NAD-dependent methanol dehydrogenase in *Bemisia tabaci*. (A) Relative gene expression between resistant (TH-2000) and susceptible (TH-S) *Bemisia tabaci* adults. (B) Relative gene expression at different developmental stages including egg, 3^rd^ instar larvae, and two-day-old unmated adult females. (C) Relative gene expression among different tissues including head, thorax, abdomen, and wing of two-day-old unmated adult females. High quality figures are available online.

The qRT-PCR results from the randomly selected differentially expressed transcripts were, for the most part, consistent with the SSH results (Figure 2, Supplementary Tables 1 and 2). For the 11 up-regulated genes, 9 of them were over-expressed in the resistant *B. tabaci*, whereas 4 out of 5 down-regulated genes were under-expressed. However, only 50% of the genes exhibited significantly different expression profiles between resistant and susceptible whiteflies (Figure 2). Most notably, F-TH_SS_58, a putative NADdependent methanol dehydrogenase EST, was over-expressed ∼12-fold in the resistant TH-2000 whiteflies in comparison to the susceptible TH-S strains (Figure 2, Supplementary Table 1). To characterize this newly identified *B. tabaci* dehydrogenase gene, its expression profiles at different developmental stages and different tissues were examined (Figure 3). In general, the transcript level of this gene was much higher in the resistant whiteflies (Figure 3A), was most abundant in egg stage (Figure 3B), and was much higher in the abdomen of adult female than in any other tissues (Figure 3C).

## Discussion

Thiamethoxam, a second-generation neonicotinoid insecticide (Maienfisch *et al*. 2001), has been used extensively for the sustainable management of *B. tabaci* in horticultural and other cropping systems (Nauen and Denholm 2005). However, like many other neonicotinoid insecticides, *B. tabaci* has developed a high degree of resistance to thiamethoxam under the laboratory selection (Feng *et al*. 2008, 2010), as well as in the field (Elbert and Nauen 2000; Horowitz *et al*. 2004; Wang *et al*. 2010) in the past decade. The molecular mechanism governing the thiamethoxam resistance in *B. tabaci*, however, has yet to be fully understood. In this study, the molecular basis of thiamethoxam resistance in *B. tabaci* was investigated using the SSH cDNA library approach. About 72 and 52 differentially expressed ESTs were obtained from forward and reverse libraries, respectively, representing up-regulated and down-regulated genes. The differentially expressed genes between the thiamethoxam-resistant and susceptible *B. tabaci* include, but not limit to, cell communication, response to abiotic stimulus, response to stress, lipid particle, nuclear envelope, cell proliferation, and nutrient reservoir activity, etc. The accuracy of the SSH method was partially confirmed by the qRT-PCR analysis, with only 50% of the randomly selected ESTs showed significant differences. Similar to microarray analysis, RNA-based SSH method tends to generate false positives. Consequently, results from both analyses need to be validated by qRTPCR.

Previous mechanistic studies suggested that neonicotinoid resistance could be associated with enhanced metabolic detoxification by cytochrome P450 monooxygenases (Zhao *et al*. 2000; Rauch and Nauen 2003; Honda *et al*. 2006; Karunker *et al.*, 2008; Wang *et al.*, 2009). In this study, only one P450-like EST (F-TH_SS_19), that has the highest similarity with a tobacco cytochrome P-450-like gene (Accession No. BAA10929.1), was significantly over-expressed in the resistant whiteflies (1.50 ± 0.05, p<0.01, Table 2). Due to the limited resolution and coverage of this SSH method, it is not uncommon that some of the genes potentially involved in the thiamethoxam resistance in whiteflies were not included. It is worth noting, however, that a NAD-dependent methanol dehydrogenaselike EST from *B.tabaci* was substantially overexpressed in the resistant whiteflies (11.56 ± 0.57, p<0.01, Table 2).

Dehydrogenases including farnesol dehydrogenase, succinic semialdehyde dehydrogenase (SSADH), aldehyde dehydrogenase, glutamate dehydrogenase, and methanol dehydrogenase can oxidize a substrate by transferring one or more hydrides (H^-^) to an acceptor, usually NAD^+^/NADP^+^ or a flavin coenzyme such as FAD or FMN. They are involved in various physiological and biochemical processes. In mammals, SSADH is thought to be responsible for the degradation of the inhibitory neurotransmitter GABA in the central nervous system (Blaner and Churchich 1979; Chambliss *et al*. 1995). SSADH homologues have been cloned and expressed in the parasitic insects *Lucilia cuprina* and *Ctenocephalides felis* (Rothacker *et al*. 2008). In addition, NADP^+^-dependent farnesol dehydrogenase was found to be involved in the juvenile hormone synthesis in mosquito (Mayoral *et al*. 2009). The NADdependent methanol dehydrogenase found in this study shed new light on the molecular understanding of thiamethoxam resistance in whiteflies. Based on these results, future studies involving cloning and functional characterization of this NAD-dependent methanol dehydrogenase are warranted to elucidate its role in the whitefly thiamethoxam resistance.

**Table 1.**
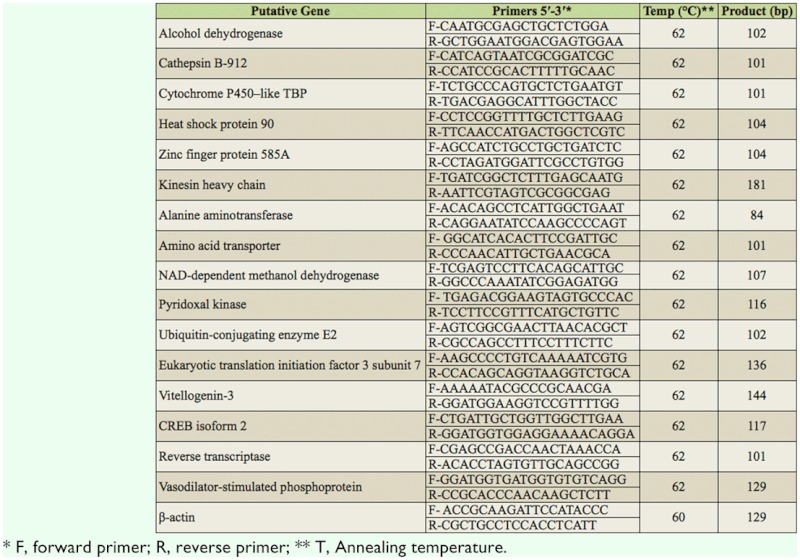
Primers used for the quantitative real-time PCR analysis.

Supplementary Table 1. Up-regulated genes identified in the SSH forward library.
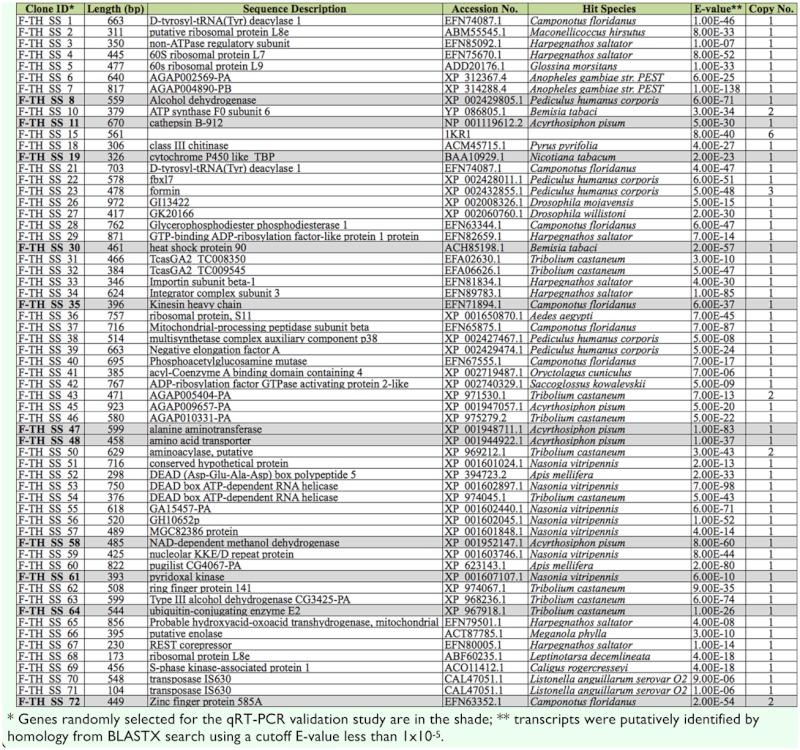

Supplementary Table 2. Down-regulated genes identified in the SSH reverse library.
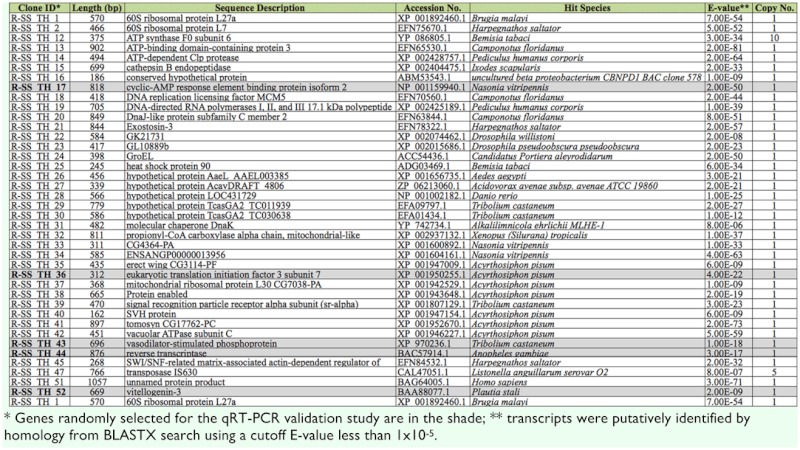

## Abbreviations:

**EST**,: expressed sequence tag;
**SSH**,: suppression subtractive hybridization;
**qRT-PCR**,: quantitative real-time PCR
